# Different CT perfusion algorithms in the detection of delayed cerebral ischemia after aneurysmal subarachnoid hemorrhage

**DOI:** 10.1007/s00234-015-1486-8

**Published:** 2015-01-23

**Authors:** Charlotte H. P. Cremers, Jan Willem Dankbaar, Mervyn D. I. Vergouwen, Pieter C. Vos, Edwin Bennink, Gabriel J. E. Rinkel, Birgitta K. Velthuis, Irene C. van der Schaaf

**Affiliations:** 1 Department of Neurology and Neurosurgery, Room G03.232, Brain Center Rudolf Magnus Department of Neurology and Neurosurgery, University Medical Center Utrecht, PO Box 85500, 3508 GA Utrecht, The Netherlands; 2 Department of Radiology, University Medical Center Utrecht, Utrecht, The Netherlands; 3 Image Sciences Institute, University Medical Center Utrecht, Utrecht, The Netherlands

**Keywords:** Subarachnoid hemorrhage, CT perfusion, Delayed cerebral ischemia

## Abstract

**Introduction:**

Tracer delay-sensitive perfusion algorithms in CT perfusion (CTP) result in an overestimation of the extent of ischemia in thromboembolic stroke. In diagnosing delayed cerebral ischemia (DCI) after aneurysmal subarachnoid hemorrhage (aSAH), delayed arrival of contrast due to vasospasm may also overestimate the extent of ischemia. We investigated the diagnostic accuracy of tracer delay-sensitive and tracer delay-insensitive algorithms for detecting DCI.

**Methods:**

From a prospectively collected series of aSAH patients admitted between 2007–2011, we included patients with any clinical deterioration other than rebleeding within 21 days after SAH who underwent NCCT/CTP/CTA imaging. Causes of clinical deterioration were categorized into DCI and no DCI. CTP maps were calculated with tracer delay-sensitive and tracer delay-insensitive algorithms and were visually assessed for the presence of perfusion deficits by two independent observers with different levels of experience. The diagnostic value of both algorithms was calculated for both observers.

**Results:**

Seventy-one patients were included. For the experienced observer, the positive predictive values (PPVs) were 0.67 for the delay-sensitive and 0.66 for the delay-insensitive algorithm, and the negative predictive values (NPVs) were 0.73 and 0.74. For the less experienced observer, PPVs were 0.60 for both algorithms, and NPVs were 0.66 for the delay-sensitive and 0.63 for the delay-insensitive algorithm.

**Conclusion:**

Test characteristics are comparable for tracer delay-sensitive and tracer delay-insensitive algorithms for the visual assessment of CTP in diagnosing DCI. This indicates that both algorithms can be used for this purpose.

## Introduction

In patients who deteriorate within the first weeks after aneurysmal subarachnoid hemorrhage (aSAH), it can be difficult to differentiate delayed cerebral ischemia (DCI) from other causes of deterioration. The use of CT perfusion (CTP) can be helpful to make this differentiation [1]. Perfusion deficits on CTP have been associated with DCI in patients with aSAH [2, 3]. An arterial input function is required to calculate quantitative perfusion values with CTP. The arterial input function can be influenced by pathology of the brain and blood vessels [4]. Occurrence of delay or dispersion between the site of the arterial input function selection and the area of perfusion measurement leads to an underestimation of cerebral blood flow (CBF) and overestimation of the mean transit time (MTT) when a delay-sensitive method is used [5]. This delay and dispersion can be caused by blood vessel occlusion or narrowing and collateral blood supply. In patients with thromboembolic stroke, the bias in the estimated perfusion parameters due to the use of tracer delay-sensitive perfusion algorithms results in an overestimation of the extent of ischemia [5–8]. Similarly, in patients with aSAH, vasospasm may cause both delayed arrival and dispersion of contrast to certain regions of the brain. As a result, the presence of vasospasm could result in overestimation of the extent of DCI when using tracer delay-sensitive perfusion algorithms. An alternative method to tracer delay-sensitive perfusion algorithms is a tracer delay-insensitive algorithm. These delay-insensitive algorithms correlate well with the final infarct areas in ischemic stroke patients [7].

The purpose of this study was to compare the diagnostic accuracy of two CTP algorithms (tracer delay sensitive and tracer delay insensitive) to differentiate DCI from other causes of clinical deterioration in aSAH patients.

## Materials and methods

### Design

Patients were retrieved from a prospectively collected series of aSAH patients admitted to the University Medical Center Utrecht between August 2007 and February 2011. In our institution all patients with aSAH routinely undergo non-contrast CT (NCCT), CTP, and CT angiography (CTA) at admission and at any time of clinical deterioration after aSAH, unless there are contraindications for CT with contrast, such as impaired renal function. Inclusion criteria for this study were as follows: (1) 18 years of age or older, (2) clinical deterioration other than rebleeding within 21 days after aSAH, and (3) follow-up imaging (CT or MR) at least 3 days after clinical deterioration. Patients were excluded in case of movement artefacts or technical failure preventing proper interpretation of the CTP scan. The study was approved by the institutional review board.

### Outcome DCI

Causes of clinical deterioration were categorized into DCI and non-DCI. DCI was defined as a clinical deterioration (new focal deficit, decreased Glasgow Coma Scale of at least two points, or both) lasting 1 h or longer with no evidence for rebleeding or hydrocephalus on CT and no other medical explanation, such as cardiovascular or pulmonary complications, infections, or metabolic disturbances [9]. The diagnosis of DCI was made by two observers (CHPC and MDIV) who were blinded for CTP data. All clinical data, except for CTP results, were available when evaluating the presence or absence of DCI. Patients with clinical deterioration who did not meet the criteria for DCI were included in the non-DCI group.

### CTA and CTP imaging

The imaging studies were performed on a 16-, 64-, or 128-multidetector CT scanner (Philips Mx8000 IDT 16, Philips Brilliance 16P, Philips Brilliance 64, Philips Brilliance iCT; Best, The Netherlands). For the CTP scan, 40 mL of non-ionic contrast agent (lopromide, Ultravist, 300 mg iodine/mL, Schering, Berlin, Germany) was injected into the cubital vein (18-gauge needle) at a rate of 5 mL/s followed by a 40-mL saline flush at a rate of 5 mL/s using a dual power injector (Stellant Dual CT injector, Medrad Europe BV, Beek, The Netherlands). The following parameters were used: 16 slice, 90 kVp, 150 mAs, 8 × 3 mm collimation; 64 slice, 80 kVp, 150 mAs, 64 × 0.625 mm collimation; and 128 slice, 80 kVp, 150 mAs, 128 × 0.625 mm collimation. For all scanners, one image was acquired per 2 s from initiation of contrast injection during 60 s, with a 512 × 512 matrix, a field of view ranging from 160 to 220 mm, UB filter and standard resolution. Depending on the multidetector-type CT scanner, for the CTA scan, another 60–80 mL of contrast was injected at a rate of 5 mL/s, followed by a 40-mL saline flush at a rate of 5 mL/s. Imaging was performed with the following: 80–120 kVp, 150–300 mAs, 512 × 512 matrix, 160–200-mm field of view, 0.9–1-mm slice thickness, and 0.45–0.5-mm reconstruction increment.

### CTP post-processing

All CTP scans were reconstructed to 5-mm slices and corrected for axial rotation and translation. For noise reduction, a filter using the time-intensity profile similarity to reduce noise in the spatial domain was applied [10]. Perfusion data were analyzed using an open-source software package called Perfusion Mismatch Analyzer (version 3.4.0.6, ASIST, Japan). The arterial input function was automatically selected by Perfusion Mismatch Analyzer and manually corrected if the automatic selection failed. An in-house software tool was developed using MeVisLab (MeVisLab®, software for medical image processing and visualization; http://www.mevislab.de) for visualization of the perfusion maps. Two different CTP algorithms were used to calculate quantitative cerebral perfusion maps for four different perfusion parameters: CBF, cerebral blood volume (CBV), MTT, and time-to-peak (TTP). The two algorithms were (1) first moment MTT and (2) block-circulant singular value decomposition. The first moment MTT determines the first moment of the time attenuation curves without using a tissue residue function and is delay sensitive [11], while block-circulant singular value decomposition is delay insensitive [12].

### CTP evaluation

Anonymized CTP color maps (CBF, CBV, MTT, and TTP) were visually assessed by two independent observers (JWD and CHPC). The observers were blinded for any other NCCT, CTP, or CTA studies but had knowledge of the patient’s clinical condition (GCS-score and focal deficit if applicable). The first observer (JWD) was an experienced observer of CTP imaging (6 years experience with CTP in DCI), while the second observer (CHPC) was a less experienced observer (1.5 years experience). For the second observer, the time between the diagnosing of DCI based on the clinical definition and the evaluation of the CTP was more than 3 months. In every patient, both CTP algorithms were displayed separately as a collection of the four perfusion maps (CBF, CBV, MTT, and TTP) and the NCCT. The two different CTP algorithms were offered randomly to each observer. For each CTP algorithm, the observers had to first indicate if a perfusion deficit was visible on all maps together, and next if it was visible on every separate CTP map. Positive findings were hypoperfused areas (lower CBF or CBV or higher MTT or TTP), indicating some degree of ischemia, that were not localized in the neurosurgical trajectory or directly surrounding an intracerebral hematoma.

### Vasospasm

The degree of vasospasm on CTA was assessed on a Philips workstation by one observer (JWD) blinded for clinical data and other imaging studies (except for the admission CTA). The vessel diameter on the follow-up scan was compared to the admission scan to assess the presence and degree of vasospasm for the following vessel segments: the proximal and distal segments of the anterior, middle, and posterior cerebral artery and basilar artery (A1, A2, M1, M2, P1, and P2 segments on both sites and basilar artery). Vasospasm was categorized as none (0–25 % decrease in vessel diameter), moderate (25–50 % decrease in vessel diameter), or severe (>50 % decrease in vessel diameter). For each patient, the vessel with the most severe spasm was noted for subgroup analysis.

### Analyses

Sensitivity, specificity, positive predictive value (PPV), and negative predictive value (NPV) for detection of DCI were calculated for both CTP algorithms and for both observers. DCI was diagnosed according to predefined criteria as mentioned above and used as the gold standard in our analyses. Since we hypothesized that vasospasm potentially affected the results of both CTP algorithms, we performed a subgroup analysis in which only patients with severe vasospasm were included. For all test characteristics, we calculated 95 % CIs.

## Results

In total, 73 patients met the inclusion criteria. Two patients were excluded because of insufficient CTP quality due to movement artefacts. Of the remaining 71 patients, 33 patients had clinical deterioration due to DCI and 38 patients (non-DCI group) had clinical deterioration from another cause (Table 1). The distribution of the patients over the different types of scanners (16-, 64-, and 128-detector scanner) was comparable in the DCI group (12, 55, and 33 %, respectively) and non-DCI group (5, 66, and 29 %, respectively). The CTA scan was assessable to evaluate vasospasm in 67 of the 71 included patients. Twenty-six of these 67 patients (39 %) had severe vasospasm.

**Table 1:** Characteristics of the 71 patients with aSAH and a clinical deterioration after admission

	Deterioration due to DCI (*n* = 33)	Deterioration from another cause (*n* = 38)
Women (*n*, %)	26 (79 %)	30 (79 %)
Age (mean, range)	54.7 (28–81)	60.9 (39–80)
Admission WFNS score (%)		
I	11 (33 %)	13 (34 %)
II	10 (30 %)	9 (24 %)
III	3 (9 %)	2 (5 %)
IV	5 (15 %)	11 (29 %)
V	4 (12 %)	3 (8 %)
Aneurysm location (%)		
Anterior communicating artery	15 (46 %)	10 (26 %)
Anterior cerebral artery	1 (3 %)	2 (5 %)
Middle cerebral artery	0 (0 %)	9 (24 %)
Posterior communicating artery	7 (21 %)	6 (16 %)
Basilar artery	5 (15 %)	7 (18 %)
Internal carotid artery	5 (15 %)	4 (11 %)
Treatment (%)		
Coil	19 (58 %)	12 (32 %)
Clip	12 (36 %)	24 (63 %)
None	1 (3 %)	2 (5 %)
Both	1 (3 %)	0 (0 %)
Vasospasm (%)		
CTA not assessable	1 (3 %)	3 (8 %)
None (0–25 %)	8 (24 %)	17 (45 %)
Moderate (25–50 %)	6 (18 %)	10 (26 %)
Severe (>50 %)	18 (55 %)	8 (21 %)

*DCI* delayed cerebral ischemia, *aSAH* aneurysmal subarachnoid hemorrhage, *n* number, *WFNS* World Federation of Neurological Surgeons, *CTA* CT angiography

### First (experienced) observer

**Fig. 1:**
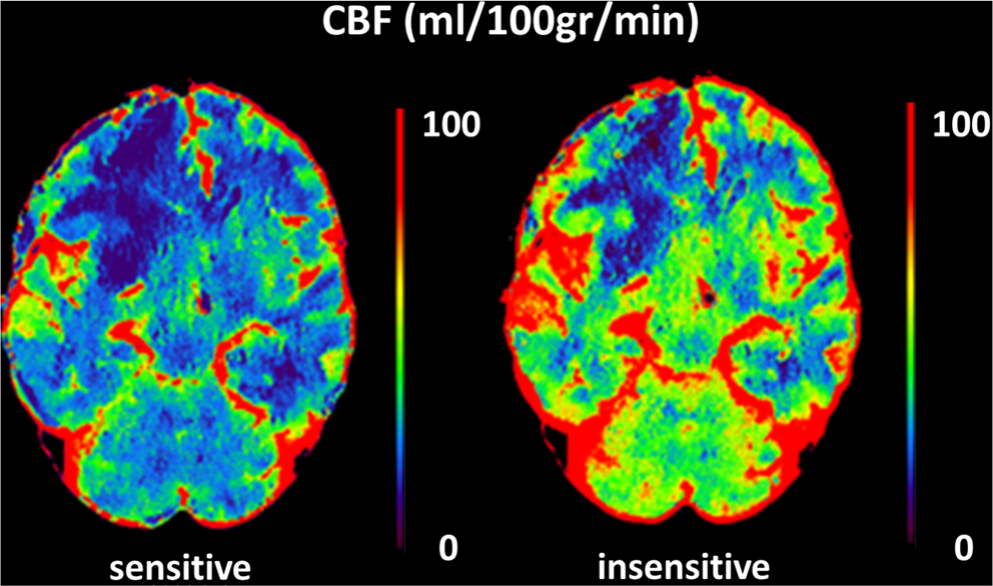
Example of a perfusion deficit on CBF maps in the right frontal lobe in an aSAH patient with DCI. Although the quantitative values are different for the delay-sensitive (*left*) and delay-insensitive (*right*) algorithms, the size of the lesion is similar

For diagnosing DCI with the delay-sensitive algorithm, the sensitivity was 0.73, specificity 0.67, PPV 0.67, and NPV 0.73. Using the delay-insensitive algorithm, the sensitivity was 0.72, specificity 0.68, PPV 0.66, and NPV 0.74 (Table 2). For the delay-sensitive algorithm, there were 24 patients with true-positive perfusion deficits (a positive finding on CTP in patients with a diagnosis of clinical deterioration due to DCI). These perfusion deficits were mostly seen on MTT (24 patients, 100 %), followed by TTP (22 patients, 92 %), CBF (12 patients, 50 %), and CBV (10 patients, 42 %). Of the 23 patients with true-positive perfusion deficits on the delay-insensitive algorithm, most deficits were seen on MTT (19 patients, 83 %), followed by TTP (18 patients, 78 %), CBF (17 patients, 74 %), and CBV (14 patients, 61 %). Figure 1 shows an example of a perfusion deficit for both the delay-sensitive and delay-insensitive algorithms.

**Table 2:** Test characteristics of visual CT perfusion assessment for diagnosing DCI

	Tracer delay	Sensitivity (95 % CI)	Specificity (95 % CI)	PPV (95 % CI)	NPV (95 % CI)
Observer 1 (experienced)	Sensitive^a^	0.73 (0.55–0.87)	0.67 (0.49–0.81)	0.67 (0.49–0.81)	0.73 (0.55–0.87)
	Insensitive^b^	0.72 (0.53–0.86)	0.68 (0.50–0.82)	0.66 (0.48–0.81)	0.74 (0.56–0.87)
Observer 2 (less experienced)	Sensitive^a^	0.64 (0.45–0.80)	0.62 (0.45–0.78)	0.60 (0.42–0.76)	0.66 (0.48–0.81)
	Insensitive^b^	0.55 (0.36–0.72)	0.68 (0.50–0.82)	0.60 (0.41–0.77)	0.63 (0.46–0.77)

*DCI* delayed cerebral ischemia, *95* % *CI* 95 % confidence interval, *PPV* positive predictive value, *NPV* negative predictive value

^a^Tracer delay sensitive is first moment mean transit time (fMTT)

^b^Tracer delay insensitive is block-circulant singular value decomposition (bSVD)

### Second (less experienced) observer

For diagnosing DCI with the delay-sensitive algorithm, the sensitivity was 0.64, specificity 0.62, PPV 0.60, and NPV 0.66. For the delay-insensitive algorithm, the sensitivity was 0.55, specificity 0.68, PPV 0.60, and NPV 0.63 (Table 2). Of the 21 patients with true-positive perfusion deficits seen on the delay-sensitive algorithm, most were visible on MTT (20 patients, 95 %), thereafter on TTP (19 patients, 90 %), on CBF (7 patients, 33 %), and on CBV (4 patients, 19 %). For the delay-insensitive algorithm, there were 18 patients with true-positive perfusion deficits, which were seen on MTT and TTP in 18 patients (100 %), on CBF in 6 patients (33 %), and on CBV in 4 patients (22 %).

### Subgroup analysis: patients with severe vasospasm on CTA

In the analysis including only patients with severe vasospasm (*n* = 26), sensitivity, specificity, PPV, and NPV also did not significantly differ between the tracer delay-sensitive and tracer delay-insensitive algorithms (Table 3).

**Table 3:** Test characteristics of visual CT perfusion assessment for diagnosing DCI in patients with severe vasospasm (*n* = 26)

	Tracer delay	Sensitivity (95 % CI)	Specificity (95 % CI)	PPV (95 % CI)	NPV (95 % CI)
Observer 1 (experienced)	Sensitive^a^	0.94 (0.73 to 1.00)	0.50 (0.16 to 0.84)	0.81 (0.58 to 0.95)	0.80 (0.28 to 1.00)
	Insensitive^b^	0.82 (0.57 to 0.96)	0.50 (0.16 to 0.84)	0.78 (0.52 to 0.94)	0.57 (0.18 to 0.90)
Observer 2 (less experienced)	Sensitive^a^	0.78 (0.52 to 0.94)	0.38 (0.09 to 0.76)	0.74 (0.49 to 0.91)	0.43 (0.10 to 0.82)
	Insensitive^b^	0.78 (0.52 to 0.94)	0.43 (0.10 to 0.82)	0.78 (0.52 to 0.94)	0.43 (0.10 to 0.82)

*DCI* delayed cerebral ischemia, *95* % *CI* 95 % confidence interval, *PPV* positive predictive value, *NPV* negative predictive value

^a^Tracer delay sensitive is first moment mean transit time (fMTT)

^b^Tracer delay insensitive is block-circulant singular value decomposition (bSVD)

## Discussion

Test characteristics for diagnosing DCI by means of visual CTP assessment are comparable for tracer delay-sensitive and tracer delay-insensitive CTP algorithms. This applies to both sensitivity and specificity as well as PPV and NPV.

To our knowledge, no other studies have compared tracer delay-sensitive and tracer delay-insensitive CTP algorithms in diagnosing DCI in aSAH patients. A study in ten patients with thromboembolic stroke found that in visual CTP assessment tracer delay-sensitive algorithms overestimated the final infarct area, while tracer delay-insensitive algorithms correlated well with the final infarct area [7]. Another study in 20 patients with unilateral steno-occlusive lesions described the correlation between different CTP parameters (both quantitative and semiquantitative) and single photon emission computed tomography (SPECT) and found that tracer delay-insensitive CTP algorithms were superior compared to tracer delay-sensitive algorithms for reliable assessment of perfusion abnormalities [13]. Our results in patients with aSAH are in contrast with these two previous studies in patients with ischemic stroke or steno-occlusive disease. An explanation might be that the delay or dispersion caused by vasospasm is not comparable to that of steno-occlusive disease or thromboembolic stroke, and only complete occlusion or stenosis >70 % leads to differences between tracer delay-sensitive and tracer delay-insensitive CTP algorithms. This is in concordance with a phantom model study which found that only a stenosis of ≥70 % resulted in a decreased outflow [14]. However, the subgroup analysis we performed in patients with severe vasospasm also did not show a difference between the delay-sensitive and delay-insensitive algorithms. This may be due the small number of patients in this analysis. Another reason could be that the difference in perfusion values between the two CTP algorithms is too small to be visually detected in the setting of DCI in aSAH patients.

We found somewhat lower test characteristics in this study compared to the test characteristics in other studies on visual CTP assessment for evaluation of DCI [1–3, 15]. In the literature, sensitivity for visual CTP assessment varies from 80 to 84 % and specificity varies from 67 to 83 % [1–3, 15]. This difference might partially be explained by the fact that in our study the evaluation of CTP was blinded for the scans that were performed earlier during admission, whereas in clinical practice all previous scans are often used in the diagnostic process. In our study, a total of five patients showed a false-positive perfusion abnormality. However, when evaluating the imaging studies performed prior to the clinical deterioration, a pre-existing infarction was already visible in the region falsely identified as a DCI-related perfusion deficit in all five patients. In clinical practice, earlier imaging studies are usually available when evaluating the presence of DCI on the CTP, and this allows discrimination of new ischemic lesions from pre-existing lesions.

In this study, the experienced observer showed a better visual discriminative performance compared to the less experienced observer. A plausible explanation could be that there is a learning curve for the visual assessment of DCI on CTP. Besides DCI, hydrocephalus, edema, intracerebral hemorrhage, or craniotomy can also cause perfusion deficits. This implies that training is necessary to interpret CTP in patients with suspected DCI. CTP interpretation could also be improved by a standardized method to determine DCI with CTP as well as better definitions of perfusion thresholds to differentiate between patients with DCI and patients without DCI [1].

A potential limitation of this study is that the diagnosis of clinical deterioration due to DCI is a diagnosis per exclusionem and subject to some misclassification. The diagnosis of clinical deterioration due to DCI is currently based on a decrease in the level of consciousness or the development of focal neurological deficits, after exclusion of other causes for the clinical deterioration. We used a definition recently proposed by an international multidisciplinary research group as the reference standard [9]. Although this is a widely adopted definition for DCI, the chance for some misclassification remains. However, because this is a random misclassification, the impact on the difference between the two algorithms will be limited.

The comparable test characteristics we found for tracer delay-sensitive and tracer delay-insensitive CTP algorithms in differentiating DCI patients from patients with other causes of deterioration indicates that both algorithms can be used for qualitative analysis in patients with aSAH. Because of the superiority of the time-insensitive perfusion algorithm in steno-occlusive stroke patients, it is likely that this algorithm will be implemented in the future. Our results show that implementation of this algorithm has no negative effects if used for patients with DCI. Our results also imply that the findings of previous studies studying DCI with delay-sensitive CTP are still generalizable and useful without an increased rate of false-positive results. The difference found between the two observers in the visual assessment of CTP implies that further research on the quality of CTP evaluation by observers with different levels of experience is needed.

To conclude, tracer delay-sensitive and tracer delay-insensitive CTP algorithms have similar test characteristics for the differentiation of aSAH patients with DCI from aSAH patients with other causes of deterioration. Both algorithms can therefore be used for qualitative evaluation of DCI in patients with aSAH.
